# Construction and Validation of a Recurrent Risk Nomogram Model for Non-Small Cell Lung Cancer within 1 Year after Radical Resection

**DOI:** 10.1155/2022/8967162

**Published:** 2022-07-19

**Authors:** Dechuang Zeng, Xiqiang Tang, Feng Nong, Jinyuan Yi, Yuanxi Yao, Shiguan Luo

**Affiliations:** Department of Cardiothoracic Vascular Surgery, Affiliated Hospital of Youjiang Medical University for Nationalities of Baise, Guangxi 533000, China

## Abstract

**Objective:**

To explore the risk factors of recurrence within 1 year after radical resection of non-small cell lung cancer (NSCLC) and construct the nomogram model.

**Methods:**

The clinical data of 186 patients with NSCLC treated with radical surgery in Affiliated Hospital of Youjiang Medical University for Nationalities of Baise were retrospectively analyzed. Multivariate logistic regression was applied to analyze the risk factors of recurrence within 1 year after radical resection of NSCLC. The R language (R 4.0.3 software package) was used in constructing the nomogram model, and the predictive value of the model was evaluated.

**Results:**

The recurrence rate of 186 patients within 1 year after radical surgery was 29.57%. After multivariate logistic regression analysis, pathological stage, number of lymph node metastasis, chronic obstructive pulmonary disease (COPD), postoperative plasma D-dimer, and carcinoembryonic antigen were independent factors for recurrence within 1 year after radical resection of NSCLC (*P* < 0.05). Based on the above independent risk factors, a nomogram model was established, with the distinction of AUC = 0.891 (95% CI: 0.819–0.964) and sensitivity and specificity of 70.3% and 97.8%, respectively. The calibration curve was close to the ideal curve. External validation of the model showed AUC = 0.801 (95% CI: 0.674–0.928), and sensitivity and specificity were 66.7% and 84.2%, respectively.

**Conclusion:**

The recurrence of NSCLC within 1 year after radical surgery was related to a variety of factors, and the nomogram model constructed based on risk factors had good goodness of fit, calibration, consistency of prediction, and prediction efficiency.

## 1. Introduction

Lung cancer is a malignant tumor with upper morbidity and mortality in China, among which non-small cell lung cancer (NSCLC) accounts for 85% of all lung cancers, and its 5-year survival rate of patients is only 10%–12% [1]. Radical surgery was an accurate and effective method for the early treatment of NSCLC, but clinical follow-up showed that the first year after surgery was usually the high-risk stage of NSCLC recurrence, with the recurrence rate as high as 20%–50%, which seriously affects the quality of life and physical and mental health of patients [2]. At present, the pathological mechanism of postoperative recurrence of NSCLC was still in the stage of exploration, and the medical community had conducted a lot of studies on the related factors of postoperative recurrence of NSCLC. These findings suggested that postoperative chemoradiotherapy, tumor differentiation degree, tumor diameter, TNM stage, number of lymph node metastasis, and mediastinal lymph node metastasis were related to the recurrence of NSCLC after radical resection [3–5]. However, there were few reports on the risk of recurrence and the nomogram model after radical NSCLC surgery, and there was still a lack of a simple, rapid, and effective method to predict recurrence after radical NSCLC surgery. The nomogram model could visualize the results of multifactor logistic regression analysis and be able to use calibrated lines to describe the quantitative relationship between variables, making the prediction results readable [6]. Based on this, this study integrated the recurrence risk factors of NSCLC within 1 year after radical surgery and constructed a nomogram model to provide a reference for clinicians to predict the recurrence risk of NSCLC within 1 year after radical surgery more efficiently.

## 2. Materials and Methods

### 2.1. General Information

The clinical data of 186 NSCLC patients treated by Affiliated Hospital of Youjiang Medical University for Nationalities of Baise from March 2016 to March 2020 were retrospectively analyzed. Inclusion criteria are as follows: (1) diagnosis of primary NSCLC by histopathological or cytological examination [7]; (2) those who were treated with radical mastectomy; (3) 1-year recurrence follow-up that was completed normally; and (4) complete clinical data such as patient information, condition, complications, surgical records, and determination of tumor markers. Exclusion criteria are as follows: (1) complicated with serious organic damage to heart, liver, kidney, and other important organs; (2) extensive metastasis of cancer cells; (3) diseases associated with a pulmonary function such as pulmonary interstitial fibrosis, pneumonia, and atelectasis; and so on. All patients underwent radical NSCLC surgery by the same group of medical staff, and the operations were successfully completed.

### 2.2. Clinical Data Collection

Review patients' electronic medical records and collect clinical data that may cause postoperative recurrence of NSCLC, including gender, age, body mass index (BMI), family history of NSCLC, diabetes mellitus, hypertension or coronary heart disease, hypoalbuminemia, smoking, pathological type, pathological stage, N stage, T stage, lesion location, maximum diameter of lesion, degree of differentiation, number of lymph node metastases, mediastinal lymph node metastasis, surgical method, myelosuppression, anesthesia method, postoperative complications, postoperative chronic pain, postoperative pulmonary infection, surgical history of lung cancer, combined chronic obstructive pulmonary disease (COPD), epidermal growth factor receptor (EGFR) mutation status, preoperative prognostic nutrition index (PNI), operation time, intraoperative blood loss, hemoglobin, serum albumin, preoperative and postoperative plasma D-dimer, KPS score at first admission, ferritin, carcinoembryonic antigen, free prostate-specific antigen, carbohydrate antigen 19–9, and carbohydrate antigen 724.

NM staging was based on the classification of lung neoplasms published by the World Health Organization (WHO) and the International Association for the Study of Lung Cancer (IASLC), 2009, edition 7 [8]. The score was used to assess a patient's functional status, with a higher score indicating better health. PNI value was calculated as follows: PNI = serum albumin value (g/L)^+^5 × total number of peripheral blood lymphocytes (×10^9^/L). Postoperative chronic pain [9] was defined as patients' postoperative pain during cough, activity, and rest, which was assessed by the digital scoring scale NRS with a score of 1–10; ≥1 was classified as chronic pain. The forced expiratory volume at 1 second (FEV1), the first second forced expiratory volume accounts for the percentage of FVC (FEV1%), forced vital capacity (FVC), forced vital capacity as a percentage of the estimated value (FVC%), and percentage of forced expiratory volume in 1 second to forced vital capacity (FEV1/FVC%) were determined 1 week before the operation. COPD was diagnosed when FEV1/FVC% < 70% [10].

### 2.3. Diagnosis of Recurrent NSCLC and Grouping of Patients

Patients were evaluated for recurrence of NSCLC by imaging examination (such as chest x-ray examination and chest computerized tomography scan), surgical or biopsy pathology, bronchoscopy, and 1-year follow-up information [11]. The recurrence of patients within 1 year after surgery was counted, and patients with recurrent NSCLC were included in the recurrence group and those without recurrence were included in the non-recurrence group.

### 2.4. Statistical Method

The data were processed by SPSS 21.0 statistical software. The measurement data subjecting to normal distribution were represented by ($\bar{x}$ ± *s*), and a *t*-test was performed for comparison between the two groups. The count data was represented by the number of cases (percentage), and the comparison between groups was performed by chi-square test or rank-sum test. Multifactor logistic regression analysis was used for analyzing the risk factors of recurrence within 1 year after radical surgery for NSCLC. The nomogram model of recurrence within 1 year after radical surgery for NSCLC was constructed by R language (R 4.0.3 software package), and the internal verification of it was conducted by bootstrap method (self-sampling 500 times). The external verification method was as follows: the data set was randomly divided into a training set and a validation set according to 7:3. The training set was used to establish the nomogram model and multifactor logistic regression analysis, and the validation set was used to verify the prediction effect of the model. The prediction effect of the model was evaluated by concordance index (CI), calibration curve, and receiver operating characteristic (ROC) curve. *P* < 0.05 was considered statistically significant.

## 3. Results and Discussion

### 3.1. Differences in Clinical Data between Groups

Among the 186 patients in this study, 55 patients (recurrence group) had recurrence within 1 year after surgery, and 131 patients (non-recurrence group) had no recurrence, with a recurrence rate of 29.57%. The differences in age, NSCLC family history, pathological stage, number of lymph node metastasis, mediastinal lymph node metastasis, combined COPD, postoperative plasma D-dimer, and carcinoembryonic antigen between the groups were statistically significant (*P* < 0.055), while the differences in other indicators were no statistically significant (*P* > 0.05), as shown in Table 1.

### 3.2. Multivariate Logistic Regression Analysis of Recurrence of NSCLC within 1 year after Radical Surgery

The indexes *P* < 0.05 in the comparison of clinical data between the two groups were taken as the independent variable, and the recurrence of NSCLC within 1 year after radical surgery was taken as the dependent variable (0 = no, 1 = yes), and they were included in the multivariate Logistic regression model. After analysis, we found that pathological stage, number of lymph node metastases, concomitant COPD, postoperative plasma D-dimer, and carcinoembryonic antigen were independent influencing factors for recurrence of NSCLC within 1 year after radical resection (*P* < 0.05), as shown in Table 2.

### 3.3. Construction of a Nomogram Model for Recurrence Risk of NSCLC within 1 year after Radical Surgery

**Figure 1:**
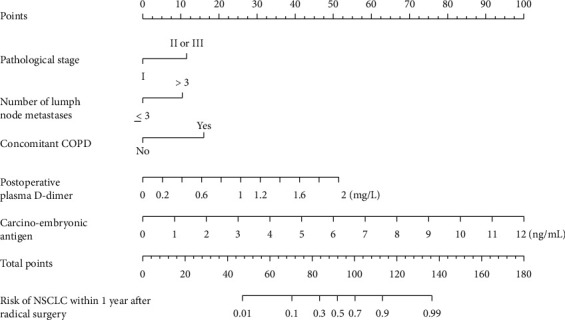
Nomogram model of recurrence risk of NSCLC within 1 year after radical resection. Note: COPD = chronic obstructive pulmonary disease and NSCLC = non-small cell lung carcinoma.

Indexes *P* < 0.05 in multivariate logistic regression analysis (pathological stage, number of lymph node metastases, concomitant COPD, postoperative plasma D-dimer, and carcinoembryonic antigen) were taken as predictive variables to construct a nomogram model of recurrence risk of NSCLC within 1 year after radical surgery, as shown in Figure 1. Nomogram prediction method: the “scoring standard” scale values corresponding to the variables of patients were added to obtain the total score, and the “recurrence risk within 1 year after radical NSCLC operation” value corresponding to the total score was the predicted risk value. If a patient's pathological stage was stage II, a number of lymph node metastasis was 4, complicated with COPD was no, postoperative plasma D-dimer was 1.2 mg/L, and carcinoembryonic antigen was 5 ng/mL; then the corresponding score and total score of each predictive variable of the patient were about: 12 + 11 + 0 + 30 + 40 = 93 points; and the corresponding risk value of 93 points was about 0.35, indicating that the probability of recurrence within 1 year after radical NSCLC surgery in this patient was about 35%.

### 3.4. Internal and External Validation of the Nomogram Model

**Figure 2:**
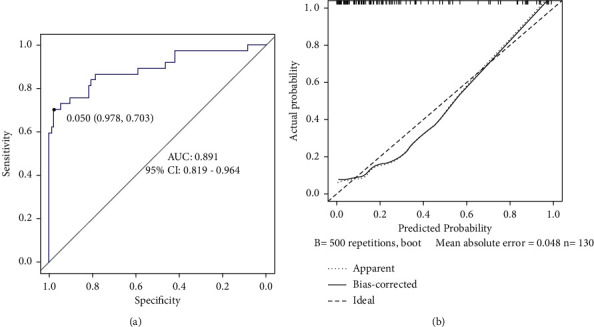
Internal validation of the nomogram model. (a) ROC curve and (b) calibration curve.

The ROC curve was used to evaluate the forecast accuracy of the model, and the calibration curve was used to evaluate the fitting degree of the predicted value and the actual value of the model. The AUC was 0.891 (95%CI: 0.819–0.964), and the sensitivity and specificity were 70.3% and 97.8%, respectively, indicating that the nomogram model had good forecast accuracy, as shown in Figure 2(a). The bootstrap method was used for internal verification of the rosette model. After repeated sampling of the original data 500 times, the calibration curve was drawn (Figure 2(b)), and the average absolute error was 0.047, suggesting that the nomogram model had good calibration degree and prediction consistency. At the same time, Hosmer–Lemeshow goodness-of-fit test *χ*^2^ = 9.854; *P*=0.275; and there was no statistical difference between the predicted risk value and the observed risk value, suggesting that the model had a good consistency with the actual risk. It showed that the model had good prediction accuracy.

**Figure 3:**
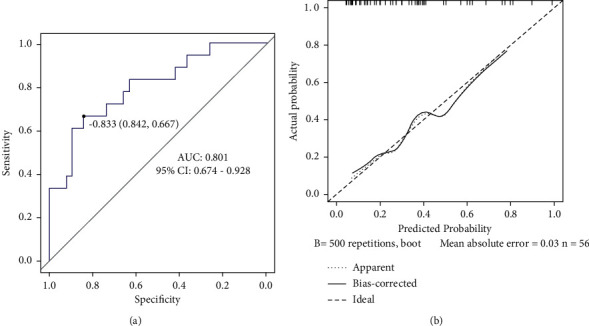
External validation of the nomogram model. (a) ROC curve and (b) calibration curve.

An external validation data set (56 cases) was used for external validation of the nomogram model. The AUC was 0.801 (95% CI: 0.674–0.928), and the sensitivity and specificity were 66.7% and 84.2%, respectively, indicating that the model had good prediction accuracy, as shown in Figure 3(a). The correction curve was close to the ideal curve, indicating that the predicted probability of the nomogram model was basically consistent with the measured value, as shown in Figure 3(b).

### 3.5. Conclusion

The reproduction rate of NSCLC tumor cells is slower, and the metastasis is later. Early NSCLC was usually treated by radical surgery, but patients were prone to relapse after surgery, resulting in difficult treatment. In this study, the recurrence rate of 186 patients within 1 year after radical surgery was 29.57%, which was lower than the results of previous research [12], which may be related to the difference in follow-up time and inclusion/exclusion criteria of subjects. Postoperative recurrence of NSCLC was affected by many factors. Integrating the risk factors of postoperative recurrence of NSCLC and constructing the nomogram model could make early prediction and intervention for patients with a high risk of postoperative recurrence of NSCLC, which was of great significance to reducing the recurrence rate and improving surgical prognosis and quality of life.

This study found that pathological stage, number of lymph node metastases, concomitant COPD, postoperative plasma D-dimer, and carcinoembryonic antigen were independent influencing factors for recurrence of NSCLC within 1 year after radical resection, which was different from previous studies. The reason may be that the higher the pathological stage was, the more serious the malignant lesions of the patients were, and the more difficult the tumor lesions were to be eradicated, leading to postoperative residual tumor cells or tissues and increasing the risk of recurrence of NSCLC. Therefore, early diagnosis and treatment of NSCLC should be strengthened to reduce the possibility of postoperative recurrence. Some researchers studied the related factors of recurrence and metastasis in patients with NSCLC after minimally invasive resection and found that stages II and III were common independent risk factors for recurrence and metastasis in patients with NSCLC after minimally invasive resection, similar to the results of this study [13]. Lymph node metastasis was the most important mode of lung tumor metastasis, which meant tumor cell proliferation and growth in patients. The more lymph node metastases there were, the more serious the damage degree to the body would be, leading to the increase in the pathological stage of patients and the increased risk of postoperative recurrence of NSCLC. The research results of Becker [14] showed that the 5-year survival rate of patients with less than 3 lymph node metastases was about 60%, which was significantly higher than that of patients with 3 or more lymph node metastases. It could be seen that a higher number of lymph node metastases tended to have a poorer prognosis. Therefore, while actively treating NSCLC in the clinic, early prevention of tumor metastasis should also be taken into account, and the possibility of postoperative recurrence should be vigilant to improve the prognosis of surgery. The increased risk of postoperative recurrence in patients with COPD may be attributed to the decline of lung function in patients with COPD, which reduced the ability of lung tissue to resist external invasion. On this basis, the activation of a large number of inflammatory factors and chemokines caused by COPD enhanced the accessibility of lung cancer. At the same time, key transcription factors (such as nuclear factor-*κ*B, transcriptional signal transducers, and activators) involved in inflammatory response promoted rapid proliferation of tumor cells and tumor angiogenesis and thus activated epithelial-mesenchymal cell transformation, leading to tumor invasion and recurrence of NSCLC [15]. D-dimer was the end product of fibrinolytic degradation catalyzed by a fibrinolytic enzyme, which could reflect the synthesis status of fibrinolytic enzyme and thrombin in the body, and the increase of its level indicated the existence of hypercoagulability and secondary fibrinolytic hyperactivity in the body [15]. The increase of plasma D-dimer after surgery suggested that the patient's coagulation and fibrinolysis system were dysfunctional, and abnormal coagulation could induce tumor recurrence [16]. In addition, D-dimer was generally low in healthy people but high in lung cancer patients, and its level increased with the increase of TNM stage [17]. The high expression of plasma D-dimer predicted the aggravation of the patient's disease, which made it difficult to achieve the eradication effect of surgery and led to an increased risk of postoperative recurrence. Carcinoembryonic antigen was an acidic glycoprotein with human embryonic antigen characteristics located on the cell membrane of cancer, which was a broad-spectrum tumor marker and an auxiliary diagnostic indicator of NSCLC. In this study, serum carcinoembryonic antigen was found to be elevated in some relapse patients, which was consistent with the results of a previous study [18], suggesting that the increased level of carcinoembryonic antigen may promote tumor invasion and cell proliferation through some effect. This study also analyzed that elevated serum carcinoembryonic antigen was an independent risk factor for recurrence of NSCLC within 1 year after radical resection, which was basically consistent with the results of the other study [19]. Another study [20] indicates that carcinoembryonic antigen was an independent predictor of postoperative recurrence of lung adenosquamous carcinoma. In conclusion, clinical attention and intervention should be intensified for patients who were at stage I or III, with 3 or more lymph node metastasis, complicated with COPD, with higher levels of plasma D-dimer (optimal cut-off value: 1.2 mg/L) or carcinoembryonic antigen (optimal cut-off value: 5.6 ng/mL), in order to reduce the risk of postoperative recurrence.

The nomogram takes advantage of calibrated lines to describe the results of multivariate logistic regression analysis and the quantitative relationships among variables, simplifying and visualizing complex predicted data, and guiding clinical risk prediction with higher efficiency. This study integrated the risk factors for recurrence of NSCLC within 1 year after radical resection (pathological stage, number of lymph node metastases, concomitant COPD, postoperative plasma D-dimer, and carcinoembryonic antigen) and constructed a nomogram model accordingly. In order to verify the prediction efficiency of the model, internal and external data were used to verify the accuracy of the model. It was found that the model had a high degree of differentiation, and the actual prediction curve was in good agreement with the ideal curve. Clinically, the sum of the scores of each risk factor could be used to predict the recurrence risk of NSCLC within 1 year after radical surgery, so as to strengthen the intervention of controllable factors. According to this nomogram model, clinical staff should attach great importance to patients with high pathological stage, a high number of lymph node metastasis, complicated COPD, high plasma D-dimer, and high carcinoembryonic antigen and take active preventive measures.

In addition, the clinicians could get the actual numerical value of the variable in the nomogram model from the patient's clinical data such as disease records, complications, and the results of tumor markers determination. Clinicians did not need to go through complex testing procedures to obtain the risk prediction results, which could be obtained only through a simple scoring reading method and addition operation after incorporating the values of predictive variables into the nomogram model. This method was simple, feasible, and effective, and would help guide clinicians to predict the risk of recurrence within 1 year after radical NSCLC surgery more efficiently, so as to make early intervention and reduce postoperative recurrence.

## 4. Conclusion

The small sample size of this study may lead to bias in the research results, which needed to be further confirmed by larger and more reliable sample size studies. Moreover, only internal verification from the center was carried out for the constructed nomogram model, lacking external verification from other centers. Therefore, the promotion and application of the nomogram model needed the support of external evidence. In conclusion, pathological stage, number of lymph node metastases, concomitant COPD, postoperative plasma D-dimer, and carcinoembryonic antigen were the risk factors for recurrence within 1 year after radical NSCLC. The nomogram model constructed based on the above risk factors had good goodness of fit, calibration, consistency of prediction, and prediction efficiency.

## Figures and Tables

**Table 1:** Differences in clinical data between groups.

Clinical data	Recurrence group (*n* = 55)	Non-recurrence group (*n* = 131)	t/*χ*^2^/Z	*P*
Gender, *n* (%)			0.502	0.479
Male	25 (45.45)	67 (51.15)		
Female	30 (54.55)	64 (48.85)		
Age, *n* (%)			19.256	＜0.001
≥60 years	28 (50.91)	25 (19.08)		
＜60 years	27 (49.09)	106 (80.92)		
BMI, *n* (%)			−0.534	0.593
＜18 kg/m^3^	10 (18.18)	20 (15.27)		
18～24 kg/m^3^	29 (52.73)	69 (52.67)		
＞24 kg/m^3^	16 (29.09)	42 (32.06)		
Family history of NSCLC, *n* (%)			4.532	0.033
Yes	10 (18.18)	9 (6.87)		
No	45 (81.82)	122 (93.13)		
Diabetes mellitus, *n* (%)			0.061	0.806
Yes	7 (12.73)	15 (11.45)		
No	48 (87.27)	116 (88.55)		
Hypertension or coronary heart disease, *n* (%)			0.889	0.346
Yes	12 (21.82)	21 (16.03)		
No	43 (78.18)	110 (83.97)		
Hypoalbuminemia, *n* (%)			1.296	0.255
Yes	10 (18.18)	34 (25.95)		
No	45 (81.82)	97 (74.05)		
Smoking, *n* (%)			0.089	0.766
Yes	21 (38.18)	47 (35.88)		
No	34 (61.82)	84 (64.12)		
Pathological type, *n* (%)			−0.237	0.813
Adenocarcinoma	29 (52.73)	66 (50.38)		
Squamous carcinoma	20 (36.36)	51 (38.93)		
Other	6 (10.91)	14 (10.69)		
Pathological stage, *n* (%)			15.745	＜0.001
I	30 (54.55)	108 (82.44)		
II or III	25 (45.45)	23 (17.56)		
N stage, *n* (%)			1.042	0.307
N0	27 (49.09)	75 (57.25)		
N1 or N2	28 (50.91)	56 (42.75)		
T stage, *n* (%)			1.488	0.223
T1	24 (43.64)	70 (53.44)		
T2 or T3	31 (56.36)	61 (46.56)		
Lesion location, *n* (%)			1.578	0.209
Left lung	32 (58.18)	63 (48.09)		
Right lung	23 (41.82)	68 (51.91)		
Maximum diameter of lesion, *n* (%)			0.126	0.722
＞3 cm	38 (69.09)	87 (66.41)		
≤3 cm	17 (30.91)	44 (33.59)		
Degree of differentiation, *n* (%)			−0.297	0.766
High	15 (27.27)	38 (29.01)		
Medium	11 (20.00)	18 (13.74)		
Low	29 (52.73)	75 (57.25)		
Number of lymph node metastases, *n* (%)			14.872	＜0.001
＞3	31 (56.36)	35 (26.72)		
≤3	24 (43.64)	96 (73.28)		
Mediastinal lymph node metastasis, *n* (%)			5.677	0.017
Yes	20 (36.36)	26 (19.85)		
No	35 (63.64)	105 (80.15)		
Surgical method, *n* (%)			0.494	0.482
Partial lobectomy lung lobe	42 (76.36)	106 (80.92)		
Complete lobectomy lung lobe	13 (23.64)	25 (19.08)		
Myelosuppression, *n* (%)			0.249	0.618
Yes	24 (43.64)	52 (39.69)		
No	31 (56.36)	79 (60.31)		
Anesthesia method, *n* (%)			0.589	0.443
Local anesthesia	26 (47.27)	70 (53.44)		
Intravenous anesthesia	29 (52.73)	61 (46.56)		
Postoperative complications, *n* (%)			0.166	0.684
＞3 complications	40 (72.73)	99 (75.57)		
≤3 complications	15 (27.27)	32 (24.43)		
Postoperative chronic pain, *n* (%)			0.150	0.699
Yes	29 (52.73)	65 (49.62)		
No	26 (47.27)	66 (50.38)		
Postoperative pulmonary infection, *n* (%)			0.249	0.618
Yes	16 (29.09)	43 (32.82)		
No	39 (70.91)	88 (67.18)		
Surgical history of lung cancer, *n* (%)			1.010	0.315
Yes	5 (9.09)	19 (14.50)		
No	50 (90.91)	112 (85.50)		
Combined COPD, *n* (%)			32.666	＜0.001
Yes	21 (38.18)	7 (5.34)		
No	34 (61.82)	124 (94.66)		
EGFR mutation status, *n* (%)			−0.334	0.738
Unknown	21 (38.18)	56 (42.75)		
Wild type	17 (30.91)	34 (25.95)		
Mutant type	17 (30.91)	41 (31.30)		
Preoperative PNI (%)	51.03 ± 6.64	48.56 ± 8.56	1.911	0.058
Operation time (min)	133.46 ± 24.16	130.67 ± 26.38	0.674	0.501
Intraoperative blood loss (mL)	106.25 ± 10.48	103.58 ± 12.42	1.398	0.164
Hemoglobin (g/L)	73.21 ± 15.36	75.22 ± 10.28	1.043	0.298
Serum albumin (g/L)	37.08 ± 8.57	35.89 ± 10.35	0.751	0.454
Preoperative plasma D-dimer (mg/L)	1.74 ± 0.52	1.72 ± 0.51	0.809	0.243
Postoperative plasma D-dimer (mg/L)	1.23 ± 0.34	0.95 ± 0.32	5.346	＜0.001
KPS score at first admission (point)	81.25 ± 10.28	83.46 ± 9.27	1.436	0.153
Ferritin (ng/mL)	323.25 ± 113.48	318.26 ± 92.58	0.313	0.755
Carcinoembryonic antigen (ng/mL)	6.72 ± 2.05	4.39 ± 1.33	9.168	＜0.001
Free prostate specific antigen (ng/mL)	0.88 ± 0.27	0.92 ± 0.11	1.439	0.152
Carbohydrate antigen 19-9 (U/mL)	41.22 ± 8.36	38.58 ± 12.49	1.437	0.152
Carbohydrate antigen 724 (U/mL)	13.58 ± 3.22	12.48 ± 4.08	1.779	0.077

*Note.* BMI = body mass index, NSCLC = non-small cell lung carcinoma, COPD = chronic obstructive pulmonary disease, EGFR = epidermal growth factor receptor, PNI = prognostic nutrition index, and KPS = Karnofsky physical score.

**Table 2:** Multivariate logistic regression analysis.

Independent variable	Assignment	*B*	*SE*	wals	*P*	*OR*	95% CI	
							Lower limit	Upper limit
Age	0 = ＜60 years 1 = ≥60 years	0.732	0.543	1.818	0.178	2.079	0.717	6.027
Family history of NSCLC	0 = No, 1 = yes	−1.012	0.805	1.580	0.209	0.363	0.075	1.762
Pathological stage	0 = I 1 = II or III	3.048	1.101	7.669	0.006	21.077	2.437	182.287
Number of lymph node metastases	0 = ≤3, 1 = ＞3	1.719	0.577	8.875	0.003	5.580	1.801	17.291
Mediastinal lymph node metastasis	0 = No, 1 = yes	−2.105	1.099	3.669	0.055	0.122	0.014	1.050
Combined COPD	0 = No, 1 = yes	1.902	0.719	7.004	0.008	6.699	1.638	27.401
Postoperative plasma D-dimer	Measured value	2.451	0.767	10.212	0.001	11.600	2.580	52.157
Carcinoembryonic antigen	Measured value	0.879	0.175	25.196	＜0.001	2.409	1.709	3.397

*Note.* COPD = chronic obstructive pulmonary disease and NSCLC = non-small cell lung carcinoma.

## Data Availability

The labeled data sets used to support the findings of this study are available from the corresponding author upon request.
